# The dynamics of global R&D collaboration networks in ICT: Does China catch up with the US?

**DOI:** 10.1371/journal.pone.0237864

**Published:** 2020-09-01

**Authors:** Thomas Scherngell, Charlotte Rohde, Martina Neuländtner

**Affiliations:** 1 AIT Austrian Institute of Technology, Vienna, Austria; 2 WU Vienna University of Economics, Vienna, Austria; Universitat de Barcelona, SPAIN

## Abstract

The purpose of this study is to identify and characterize the structure and dynamics of global R&D collaboration networks in ICT by analyzing cross-country co-patents, with a special focus on the role of China. We employ a Social Network Analysis (SNA) perspective, using information on more than 77 thousand co-patents from 2001–2015. These co-patents are disaggregated by three time periods and four ICT subsectors. Global measures for the network as a whole, as well as local measures on the positioning of countries in the networks are interpreted. The empirical results are highly interesting. First, international R&D collaboration networks in ICT show a dynamic transformation in becoming larger in magnitude (more countries but also more inter-linkages), less centralized and more densely connected, though with varying degrees across ICT subsectors. Second, the powerful position of the US weakens relatively compared to other, increasingly connected countries, in particular China. While China has already surpassed the US in total patenting in ICT in 2015, China is now also catching up from a network perspective shown by its growing central position over the observed time period.

## 1 Introduction

In an increasingly globalized world, Research and Development (R&D) collaboration networks–defined as sets of organizations interacting with each other in R&D activities–hold enormous potentials and opportunities and have become an essential element for the successful generation of innovation ([1], among others). Especially, for the Information and Communication Technology (ICT) sector–a knowledge-intensive sector shaped by fast innovation and production cycles [2]–extensive networking in R&D is essential and of increasing importance, because external knowledge can be collected and integrated faster and more effectively in the innovation process. Here, the increasing competitive position of China, challenging the exclusive and traditional leading position of the US, has been highlighted as one of the most important issues defining the future global economic development since innovations in ICT are an important driver for economic growth given their potential to increase productivity in a wide range of economic sectors [3].

### 1.1 Objectives and focus of the study

We can observe an increasing body of empirical literature investigating R&D collaboration networks at different spatial and sectoral levels. These works employ different indicators to measure such networks, most prominently co-publications for scientific collaboration, co-patenting networks for collaborations in more commercially oriented, technological knowledge projects, or joint R&D projects, often publicly funded (see [4] for a recent overview). However, there are only scarce insights into how the global R&D collaboration networks have developed in ICT, and whether we can also observe a shift towards a more important role of emerging countries, in particular China. One exception is the study of [5] investigating the global ICT R&D network by mapping global R&D locations belonging to multi-national companies. Still, there is no empirical literature yet directly investigating R&D collaboration in terms of joint knowledge creation; in particular, there is no work telling us something about structures and dynamics of these networks across ICT subsectors (with telecommunication probably being considered as the most important one).

This is the gap this study contributes to, in particular, from the angle of China’s catch-up process challenging the position of the US in the global ICT industry. Accordingly, the objective is to identify and characterize the structure and dynamics of global R&D collaboration networks in ICT as a whole, and in four ICT subsectors, with specifically shifting emphasis to global structural transformations and the changing role of China in the network. We are inspired by previous research from [6] for the global pharmaceutical R&D network in terms of our empirical setting and methodological choices. We mobilize a large-scale dataset containing information on cross-country co-patents, i.e. patents that feature inventors from at least two different countries. We use patent applications in ICT from 2001 to 2015 and employ a Social Network Analysis (SNA) perspective to gain an overall perspective of cross-country R&D co-operation, and to trace the changing role of specific countries in the network.

### 1.2 Theoretical framework

In its focus and objective, the study follows the literature stream investigating structures and dynamics of R&D collaboration networks from a social network perspective. In that sense, it is embedded in theoretical debates from innovation research most directly, but also economic growth theories in the wider sense, as well as from social network theoretical considerations on network structural mechanisms and dynamics. In terms of innovation and economic growth theories, two arguments are central to this study: *First*, the importance of R&D and innovation for economic productivity growth–and accordingly, societal development as a whole–which is widely discussed in theoretical and empirical literature (see e.g., [7, 8]), constituting the overall umbrella on why we need a better understanding on the mechanisms of R&D and innovation processes.

*Second*, R&D is increasingly viewed as interactive process between researching organizations, often referred to as the increased networked character of R&D (see [1]). Here, R&D collaboration networks are considered as essential element for the successful generation of innovation, in particular in an increasingly globalized world with rapidly changing demand patterns which increases uncertainty and risk in the research and innovation process (see [9, 10]). Accordingly, empirical works have observed an increased collaboration intensity in different R&D phases, ranging from scientific collaborations reflected in joint academic publications (see, e.g., [11–18]), to more commercially oriented R&D collaborations in technological knowledge production as reflected in joint patenting activities (see, e.g. [19–22]) or technology transactions (see [23]) and, in between, pre-competitive project-based R&D collaboration (see e.g. [24–26]).

Moreover, the literature on R&D internationalization tells us that such collaborations become increasingly internationalized in times of global innovation competition and value chains (see [16, 17, 27, 28]). Sharing new knowledge as basis for innovation leads to increased cross-border R&D collaboration (see, e.g., [29] and [26] for the European case). Recent works in this context point to the evolution from a bipolar world once led by Anglo-American countries that is gradually replaced by a tri-polar world (Europe, North America, and Asia-Pacific) (see, e.g., [17]). However, the development of collaboration links does also relate to the individual economic and political framework conditions of a country.

Related to this, innovation processes become more complex, characterized by a higher variety in the combination of different pieces of knowledge coming from different technological domains (see, e.g., [1]). Therefore, innovating actors are forced to increasingly tap into external knowledge sources, and integrate external knowledge in their own research and innovation processes, usually transferred via R&D collaboration networks [9]. In the latter context, there is also an intensive debate ongoing in the literature discussing in which way such collaborations can increase the productivity of R&D (see, e.g., [30, 31] for scientific collaborations, [32] for R&D projects).

Turning to the theoretical considerations inspired from network science, it is to be stressed that real-world social networks, such as R&D collaboration networks, develop along certain network structural specific mechanisms (see e.g., [20, 33]). In terms of R&D collaboration networks, we can observe for instance, that such networks increase in density and connectedness over time due to long-lasting and sustainable ties between researching organizations (see, e.g. [11] for scientific collaborations, [22] for technological ones). Specific structural mechanisms at stake are referred to as homophily (more similar nodes attach to each other), preferential attachment (peripheral nodes tend to attach to central nodes in the network) and triadic closure (triangles of three partners in the network leading to a higher clustering). Such mechanisms have been observed in real-world R&D collaboration networks (see e.g. [22]) and may also be at stake in the global R&D collaboration network in ICT.

Moreover, the specific ICT background as one important element of the theoretical embedding of the study has to be considered when setting up the analysis and interpreting the empirical results. *First*, the relevance of ICT for the economy as a whole has been widely stressed (see e.g. [34–38]). ICT breakthroughs are often radical and disruptive innovations paving the way for completely new business models (with the internet being the most prominent one in the closing 20^th^ century, and trends in artificial intelligence the most recent one). *Second*, ICT as a general-purpose technology is generically important for many other technological fields, and more or less all economic sectors ([38, 39]). Therefore, a highly developed ICT sector as well as advanced capabilities to innovate in ICT brings countries strategic competitive advantages, and–in the mid- to long term–a leading position in the global economy [40]. *Third*, and specifically important in the context of this study, the rapid technological development of the ICT sector reinforces risks and uncertainties in innovation processes, making R&D collaboration an even more important arrangement for dealing with these risks (see, e.g. [41]). Related to this, scholars [38] stress the importance of getting a better understanding on how ICT affects economic performance. Accordingly, looking at the developing R&D collaboration networks at a global scale can be a promising complementary element. *Fourth*, in light of the specific spotlight in this study placed on the role of China in the network, previous literature provides substantial empirical evidence on the enormous increase of the Chinese innovation potential in ICT per se, as reflected by the increasing global shares in patenting and publication activities related to ICT [42, 43], peaking with China taking the first rank from the US in ICT patenting (absolute numbers) in 2015 [44]. However, whether this rise of China is also to be observed in the global R&D collaboration network in ICT is yet unknown.

The remainder of this study is organized as follows. Section 2 discusses in some more detail the data and methods used. Section 3 presents the empirical results before Section 4 closes with a discussion against the background of the global innovation competition.

## 2 Data and methods

This study uses co-patents to analyze the structure and dynamics of international R&D collaboration networks in the ICT sector, and by this, follows several previous empirical studies using co-patents as an indicator for R&D collaboration at different geographical scales, for different technological fields, and economic sectors (see [4] for an overview). Co-patents are defined as patent applications that feature at least two different *inventors*, and, in this sense, clearly indicate collaborative R&D activities between them (see, e.g., [45, 46]; note that co-patents are sometimes also defined as patents featuring two different applicants. However, this is more a business cooperation and not necessarily an indication of joint R&D). When these inventors are located in different countries, a co-patent accordingly indicates a collaboration in R&D crossing country borders.

Patents are widely used as indicator for new technological knowledge, representing the gain of new technological knowledge as direct result of invention processes (see, e.g. [47] for a discussion). Hence, in using co-patenting, we capture a very specific form of commercially oriented, technological collaborative R&D, in contrast to other indicators such as co-publications or joint projects that are more indicative of scientific collaborations. However, using patents as indicator also comprise certain limitations (see [47, 48], among many others). While cross-country comparisons can often be inflated by the fact that the patent propensity differs markedly across economic sectors [49], this limitation is minimized in this study since it focuses on ICT per se. Moreover, we consider patent applications applied for under the Patent Cooperation Treaty (PCT), refining to inventions of global relevance applied for via common procedures across countries. By this, we avoid bias related to different regulations at different national patent offices. Moreover, it is stressed that patents as indicator for R&D activities are more biased for peripheral regions, due to missing capabilities and resources (patenting is costly and requires experience and wider knowledge about state-of-art in technological fields, etc.). However, with our focus on PCT patents, we delimit our analysis per se to actors (essentially firms located in the countries) that are capable to apply for patents at a global scale (see [50]).

In this study, the data used covers a sample of about 77 thousand co-patents of the ICT sector and sub-classes from the time period 2001 to 2015 (patent data have a time lag of up to three, sometimes four years, in particular PCT patents, as they are recorded much later in the databases than they have been applied for, see [51]; here, we take the most actual year that is available and meaningful at the same time in terms of data quality). The classification of ICT patents is based on the assignment developed by the OECD [35] referring to the 8^th^ edition of the International Patent Classification (IPC). Here, ICT patents are classified into four technological fields, that are *Computers and Office Machinery*, *Consumer Electronics*, *Telecommunications* and *Measurements and Semiconductors*. Next to the technological categorization into these fields, the study divides the time frame into the periods of 2001 to 2005, 2006 to 2010 and 2011 to 2015 according to the date of the patent application filing, enabling to look at the evolution of the network, in particular the changing positions of countries. We extract data from the RISIS-Patent database (to be accessed for research under https://rcf.risis2.eu/dataset/5/metadata), a standardized version of the PATSTAT database by the European Patent Office (EPO). S1 Appendix provides a more detailed description of the processes of data extraction and preparation, specifically the assignment of patents to ICT and respective technological subfields.

Turning from data to methodology, this study lies in the vein of the literature stream that considers a social network perspective as highly useful to study international R&D collaboration (see, e.g., [4, 11, 52]). In general, Social Network Analysis (SNA) has come into fairly wide use for the analysis of social systems, offering a wide range of analytical tools disclosing the structure and dynamics of such systems [53]. While SNA measures have initially been derived to be interpreted at the individual level of socially interacting individuals, it has also come increasingly into use to analyze internationalization trends in networks of R&D collaboration across countries (see, e.g., [6, 22, 52]. This is usually done by aggregating individual level information (in our case inventors) on collaborations to the country level and shifting attention–away from the traditional variable-centric approach–to a structural-relational angle.

In our analytical approach, we initially need to formally define the network under consideration, and second, derive some respective–global and local–network analytical measures that characterize structural changes at the global scale (the network as a whole), and the local scale (the changing role of specific nodes, in our case countries). Graph theory sets out the basic mathematical framework to formally describe our global R&D collaboration network in ICT. In our case, we define a graph *G* = (*N*, *L*, *V*) with *N* = {*N*_1_,*N*_2_,…, *Ng*} being a set of nodes (here countries) which is related through a set of edges *L* = {*L*_1_,*L*_2_,…,*L*_*M*_} and a set of weights *V* = {*V*_1_,*V*_2_,…,*V*_*M*_} for each edge, in this study the number of co-patents between two countries. The topology of a graph can be decoded in a *n*-by-*n* adjacency matrix, where *n* denotes the number of nodes, in our case countries:

$$X_{t}(i,j)=\left(\begin{array}{cccc} x_{11} & x_{12} & \cdots & x_{1n}\\ x_{21} & x_{22} & \cdots & x_{2n}\\ \vdots & \vdots & \ddots & \vdots \\ x_{n1} & x_{n2} & \cdots & x_{nn} \end{array}\right)i,j=1,\ldots ,n$$ (1)

where one element of *X* corresponds to the number of joint co-patents between countries *i* and *j* at time *t*. The total number of neighbors i.e. partner countries of a node is referred to as degree of this node. With the adjacency matrix as defined by Eq (1), we can derive a number of global and local network analytical measures relevant for the purpose of this study. In global terms, we rely on the following global network measures to shed some light on whether the global structure of the ICT network changes (see Wasserman and Faust [54] for formal definitions of these measures; all SNA indicators described in this section have been calculated using the *igraph* package available in cran.r-project.org)

- *Degree centralization* is defined as the variation in the degrees of vertices divided by the maximum degree variation which is possible in a network of the same size. It characterizes the concentration of links across nodes, taking a value of 1 if it is fully concentrated on one node (star-like network) and zero if all nodes have the same degree (fully connected graph).
- *Mean degree* is defined as the sum of individual degrees divided by the number of nodes and is used as an indicator for the connectedness of a network.
- *Density* is the relation between the actual number of edges and the possible number of edges and hence, indicates how connected the network is compared to its maximum connectedness.
- *Average path length* is specified as the average number of steps along the shortest paths for all possible pairs of network nodes; a *path* is the alternating sequence of nodes and links, such that the shortest path (or geodesic distance) is defined as the number of nodes to be passed in the shortest possible path from one node to another. The average path length is a measure of efficiency of information flow, i.e. a shorter average path length is conducive for information flow in the network.
- *Clustering* is defined using the transitivity concept that is the connection of two nodes via a third node (often referred to as a ‘clique’). The more cliquish a network and the smaller the average path length, the more a network shows so-called small world characteristics [55].

Next to the global view we are interested in the local positioning of individual nodes (countries) in the network. In SNA, this can be captured by using different kinds of network centrality measures, providing information on the prominence of nodes with respect to different qualitative dimensions. Here, we rely on the degree-based centrality, betweenness centrality and eigenvector centrality (see again [54] and [6] for a formal definition).

- *Degree-based centrality* is defined by the degree of a node, i.e. the number of co-patents. It is a measure of connectedness of a single country.
- *Betweenness centrality* of a node is defined as the sum of the ratio of the shortest paths between any two nodes in a graph that pass through that node. That is, the betweenness centrality describes the importance of a country as connector (often referred to as ‘gatekeeper’ or ‘broker’) between other countries.
- *Eigenvector centrality* is defined as the degree of a node weighted by the degree the node is connected to. Therefore, it is also referred to as prestige centrality since it indicates whether a country is connected to prominent other countries (having a high degree) or to rather less connected countries.

Regarding the R&D collaboration network perspective, the described global network measures can be understood as indicators for the efficiency of knowledge diffusion and rapidness of access to new knowledge by means of collaborations (e.g. density, average path length), while local network measures indicate countries being knowledge spreaders, and/or knowledge gatekeepers (e.g. degree and betweenness centrality).

## 3 Results

In our empirical analysis, we have calculated the global and local SNA measures (as described in Section 2) for 15 networks; that is, for the ICT network as a whole, and for the four subsectors–each for the three time periods. Initially, Table 1 presents the results of the global SNA measures. Overall, the increasing importance of R&D collaborations in ICT is clearly confirmed, total and across all subsectors, as expected from theoretical considerations in innovation studies as well as from network theory (see Section 1). In total ICT, the number of collaborations almost doubled its value from about 18,000 co-patens in 2001 to 2005 up to 33,700 co-patents in 2011 to 2015. This growth is even more pronounced in the sub-industries of *Telecommunications* and *Computer*, *Office Machinery*, and points to the increased necessity but also openness for actors to engage in ICT R&D collaborations. The transformation towards more international collaboration is also shown by the global network structural measures. The density is increasing by nearly 15%, from 0.10 in the first period (2001 to 2005) to 0.13 in the latest (2011 to 2015). Also, the clustering coefficient, that indicates the connectedness of the neighbors of a country, increased considerably from 0.43 (2001–2005) to 0.50 (2011–2015), pointing to a more cliquish structure and triadic closure in the network, a phenomenon that we expect for social systems from a network theory perspective, that is also found in similar studies at an aggregated level or for other sectors (see, e.g. [6, 17]). In relation to this, the higher connectedness is also supported by the average path length between two countries that is slightly decreasing, indicating a “small-world phenomenon”, i.e. the members of a social network being connected via short paths.

**Table 1:** Global SNA indicators in ICT and ICT sub-industries (2001–2015).

	Total ICT			Consumer Electronics			Tele- communications			Computer, Office Machinery			Measurements/ Semiconductors		
	(1)	(2)	(3)	(1)	(2)	(3)	(1)	(2)	(3)	(1)	(2)	(3)	(1)	(2)	(3)
**# of nodes**	123	131	132	55	60	66	93	91	94	95	97	103	109	110	113
**# of edges**	776	923	1113	203	268	315	431	503	580	452	589	717	592	674	799
**# of links (in thousands)**	18.68	25.36	33.66	1.37	1.86	2.31	5.98	9.04	13.01	5.98	7.34	10.43	8.61	9.92	11.62
**Density**	0.10	0.11	0.13	0.14	0.15	0.15	0.10	0.12	0.13	0.10	0.13	0.14	0.10	0.11	0.13
**Clustering coefficient**	0.43	0.47	0.50	0.38	0.44	0.45	0.43	0.46	0.47	0.39	0.43	0.48	0.42	0.45	0.47
**Average path length**	2.10	2.08	2.06	2.07	2.07	2.01	2.17	2.12	2.07	2.13	1.97	2.02	2.11	2.09	2.09
**Mean degree**	12.62	14.23	16.86	7.38	8.93	9.55	9.27	11.05	12.34	9.51	12.14	13.92	10.86	12.25	14.14
**Nodes (%) w. degree higher than mean**	4.51	4.22	4.31	9.85	8.58	7.94	6.50	6.16	5.17	6.19	4.75	5.02	5.41	4.75	4.76
**Degree centralization**	0.68	0.68	0.63	0.66	0.63	0.70	0.62	0.61	0.66	0.66	0.76	0.71	0.68	0.62	0.59

(1) 2001–2005, (2) 2006–2010, (3) 2011–2015; # denotes “number”; Density is the share observed links relative to the overall possible links between all nodes; the clustering coefficient measures the number of closed triangles in the network, the average path length is the average distance between all nodes measured in terms of the edges between them. Mean degree denotes the mean number of neighbors (i.e. partner countries) each node has in the network, while degree centralization measures the concentration of links across the nodes.

Reflecting on some sector-specific differences, they are generally quite minor for the global network perspective. Some differences are interesting and therefore worth mentioning. For instance, the average path length shows a decreasing tendency for all sectors over the observed time period, except for *Computer*, *Office Machinery* with a strong decrease between the first two time periods, while then it slightly increases again until the third period. One explanation may be that this sector has been earlier in its innovation cycle (already in a more mature innovation phase), and therefore the network has started to be integrated earlier as shown by the more pronounced decrease in average path length between the first and the second time period as compared to the other sectors. Another interesting element can be pointed out in terms of the development of the degree centralization. While the centralization of the total network decreases, it slightly increases for the other sectors (except *Measurements and Semiconductors*). This shows that within the sectors, concentration on some strong countries increases, while the total (that also consists of the inter-sector collaboration) tends to be more distributed, i.e. when accounting for inter-sectoral collaboration, peripheral countries are more integrated. This could be related to the rise of a few but significant countries in the network as will be reflected further on in the local analysis that follows.

Before we turn to the results of the local SNA analysis, we take a combined look on global and local characteristics by means of some illustrative network visualizations. Here, we follow a force directed approach for network visualization taking the so-called *Yifan Hu* layout algorithm [56]. This is, countries with a high centrality are located in the center of the network visualization, and countries with a high interaction intensity and a similar structure of partnerships are located nearer to each other. We have chosen the size of the nodes to be visualized proportional to their degree, and the edges proportional to their weights, i.e. their collaboration intensity. In this sense, these visualizations are very illustrative and effective means to observe global dynamics, but also changing roles of individual countries.

**Fig 1:**
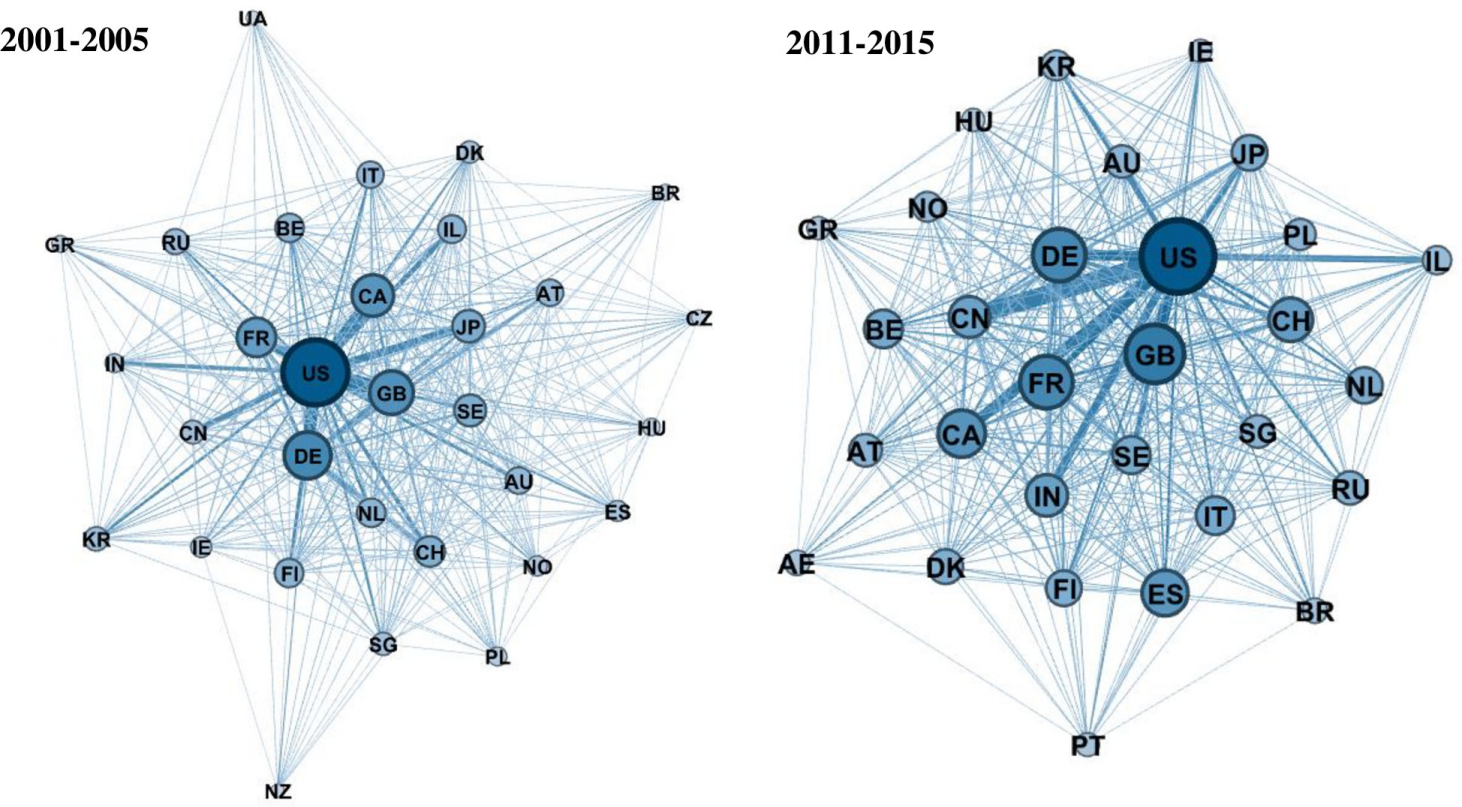
Global R&D collaboration network in ICT. Country codes given in S1 Appendix.

Fig 1 initially presents the global network visualization of ICT as a whole, comprising the earliest and the latest observed time period. Note that we have limited the visualization featuring just the top-30 countries in terms of their degree, i.e. number of collaborations (adding all nodes would lead to a severe cluttering problem). For the first time period, it can be seen that the networks have a highly connected center with relatively less connected countries in the environment which underlines the relatively high centralization measures (see Table 1), i.e. a few countries, the US being the main hub, cover most of the links. However, the network becomes clearly less “star-like” with an expanding center for the most recent time period (2011–2015), i.e. the centralization is decreasing. The countries of the network have become more densely connected, and also, peripheral countries of the outer environment become more connected to each other in the center of the network.

Turning to the subsectors (see S1 Appendix), we find not only a much higher network density and connectivity over time, but also more structural shifts. In *Telecommunications*, China has significantly increased its collaboration intensity (as shown by the size change of the node in Fig A of S1 Appendix) and comes closer to the US as central network player (though it has not been in the periphery already in the first time period as reflected by the position of the node). Also, the UK, Germany, Sweden and Finland have a high centrality in *Telecommunications*. France is less central and also Canada shows a decreasing centrality. *Computer*, *Office Machinery* and *Measurement and Semiconductors* show quite similar patterns, while in *Consumer Electronics*, the network structure as a whole and the positioning of individual countries changes to a lesser extent.

However, while the network visualizations are very insightful in illustrating the overall increasing importance and density of R&D collaboration, the interpretation of individual countries must be done with caution and hence, needs to be specifically supported by our local centrality measures as introduced in the previous section. The respective local SNA indicators are presented in Table 2, showing the ranking of the top-10 countries by their degree, eigenvector and betweenness centrality. In general, they underline quite clearly the impressions from the network visualizations. While the US is still featuring the highest centrality in all sub-industries over the whole time period observed (2001–2015), its centrality is decreasing in relative terms, i.e. other countries clearly catch-up in term of gaining a more central network positioning. This development seems of particular importance in the fields of *Telecommunications* and *Measurements and Semiconductors*. In *Consumer Electronics*, however, the US is still holding a strong central network position.

**Table 2:** Top ten centralities of countries in the global ICT network (2001–2015).

**2001–2005**					
**Country**	**Degree**	**Country**	**Eigenvector**	**Country**	**Betweenness**
**US**	**95**	**US**	**1.00**	**US**	**2667.43**
DE	67	DE	0.92	DE	742.50
GB	62	GB	0.89	GB	663.27
CA	59	CA	0.85	CA	655.42
FR	55	FR	0.82	FR	534.53
JP	45	CH	0.77	KR	339.64
SE	44	SE	0.77	SE	248.89
CH	42	JP	0.76	JP	240.45
NL	39	NL	0.75	RU	227.26
FI	39	BE	0.73	BE	204.85
**CN (18.)**	**31**	**CN (16.)**	**0.64**	**CN (13.)**	**152.14**
**2006–2010**					
**Country**	**Degree**	**Country**	**Eigenvector**	**Country**	**Betweenness**
**US**	**103**	**US**	**1.00**	**US**	**3214.10**
GB	70	DE	0.93	FR	1095.15
DE	70	GB	0.92	DE	713.40
FR	67	FR	0.87	CA	690.72
CA	62	CA	0.83	GB	6.30
SE	48	SE	0.81	DK	278.79
IT	47	CH	0.79	ES	233.80
CH	47	IT	0.79	FI	202.15
ES	46	AT	0.77	BE	195.52
**CN**	**46**	**CN**	**0.77**	IT	171.20
				**CN (12.)**	**149.42**
**2011–2015**					
**Country**	**Degree**	**Country**	**Eigenvector**	**Country**	**Betweenness**
**US**	**100**	**US**	**1.00**	**US**	**2399.79**
GB	79	GB	0.96	FR	918.56
FR	71	DE	0.94	GB	862.90
DE	71	FR	0.91	CA	563.87
CA	63	CH	0.87	JP	433.88
ES	61	ES	0.87	DE	404.68
CH	58	CA	0.86	ES	394.04
IN	55	IN	0.84	IN	377.60
**CN**	**54**	**CN**	**0.81**	RU	315.66
IT	50	BE	0.78	**CN**	**311.47**

Country codes given in the Appendix; Degree denotes the number of neighbors (i.e. partner countries) in the network; Eigenvector centrality relates the degree of a node to the degree of its neighbors (the higher, the more a node is connected to other central nodes); betweenness centrality measures the number of shortest paths in the network passing through a node (as a share to all shortest paths), expressing the ‘gatekeeper’ or ‘brokerage’ position of a node in the network.

China obviously takes a quite peculiar role that is worth to be discussed in some more detail. It is quite remarkable that China is recently just arising with a very central network position in the international R&D collaboration network in ICT, considering where it comes from in the initial time period (2001–2005). Reviewing the top-10 ranking of the total ICT network in 2011 to 2015, China is moving closer to recent big players like the US, UK, Germany and Sweden (something that has also been observed for scientific collaborations described as a shift from a bipolar to a tripolar world, see [17]). It is actually holding position nine in both, degree and eigenvector centrality, and position ten in betweenness centrality in total ICT (see Table 2). This is remarkable, given that China has been on rank 18th in the first period, climbing up 9 ranks over the observed period. The rise in betweenness centrality is even more interesting, i.e. China does not only collaborate more intensively in terms of absolute numbers, but also increasingly acts as knowledge ‘gatekeeper’ in the networks controlling knowledge flows between other countries.

These results are also quite interesting in light of other recent empirical works. In science, for instance, China has–in relative terms–increased its collaboration activities more between large Chinese cities, i.e. at an intra- rather than an international level (see [15]). In ICT and in terms of co-patenting, i.e. more industrial oriented R&D collaboration, this cannot be concluded given the high growth of international Chinese co-patents in comparison to intra-Chinese co-patents in ICT. Using the information from our dataset, international co-patents have increased by 111% between the first and the most recent time period (we find 2,209 intra-Chinese co-patents for the first time period, and 6,889 for the most recent one, while international co-patents have been 5,688 and 12,040, for these two time periods), and intra-Chinese patents by 211%. Assuming that the majority of the intra-Chinese co-patents take place within a specific city (e.g. Beijing, Shanghai or Guangzhou with its enormous number of research organisations and/or research active firms), and not between cities, one can fairly assume the inter-national level has become more significant in terms of collaboration than the inter-city level within China. However, the overall intra-Chinese collaboration intensity has increased remarkably, double as much as the international one.

**Fig 2:**
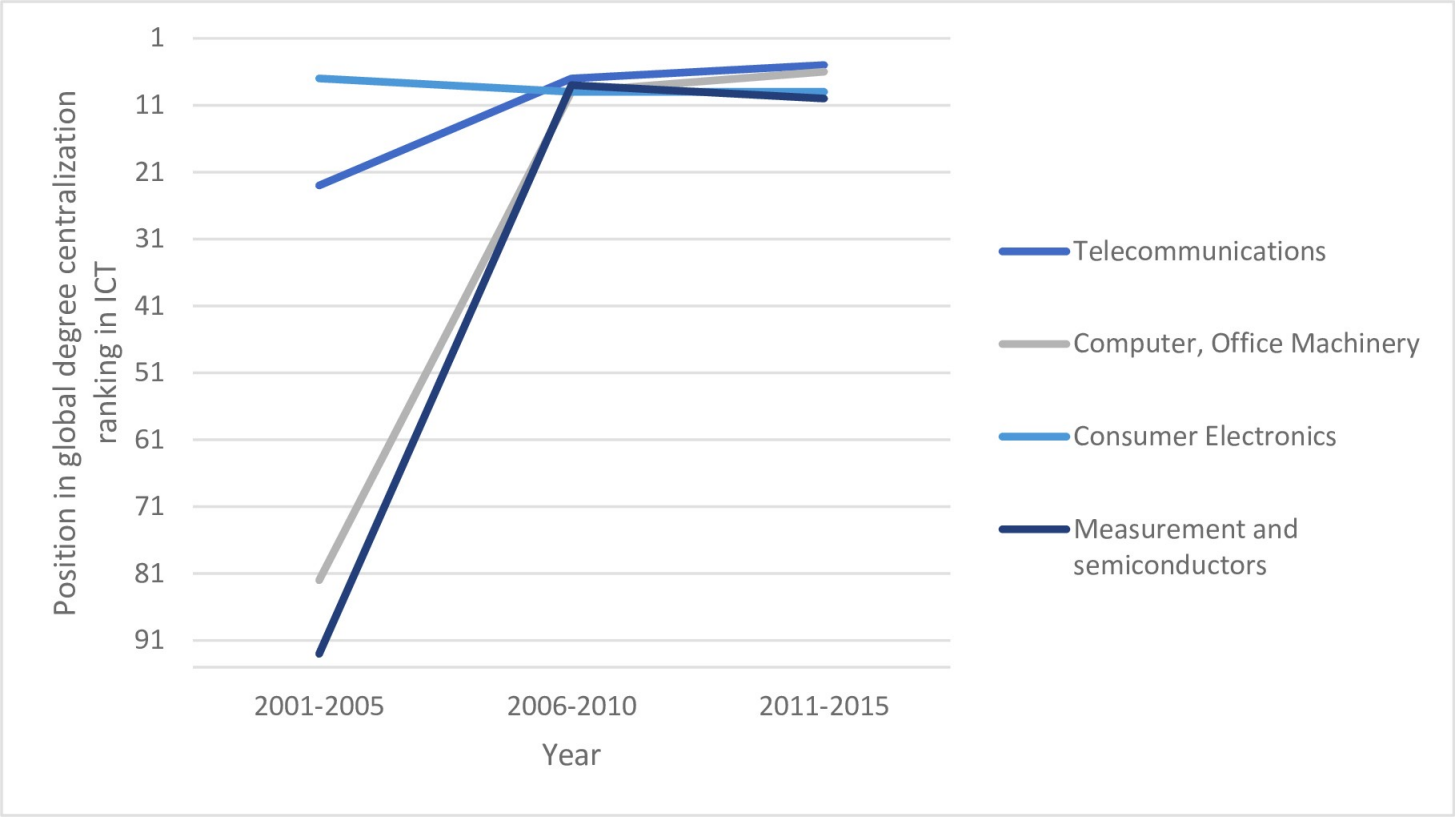
China's global ranking of degree centrality in ICT (2001–2015).

Complementing these results by looking at China´s position in the four sub-sectors, a strong development can be especially observed in *Telecommunications* and *Computer*, *Office Machinery*. This may also be significantly related to targeted innovation policy endeavors of the Chinese government in that direction (see [16]). Fig 2 illustrates the rise of China in the global ranking of degree centrality over the observed time periods in the four sub-industries of ICT. Two insights are specifically striking: First, China has advanced to a top-10 position in all sectors under consideration. Second, this has come with an immense catching-up in *Telecommunications* (rank 23 to rank 5), but even more in *Computer and Office Machinery*, and *Measurement and Semiconductors* (rank 82 to rank 6, and rank 93 to rank 10, respectively). In *Consumer Electronics*, China has slightly declined but started from a relatively high level (rank 6 to rank 9).

**Fig 3:**
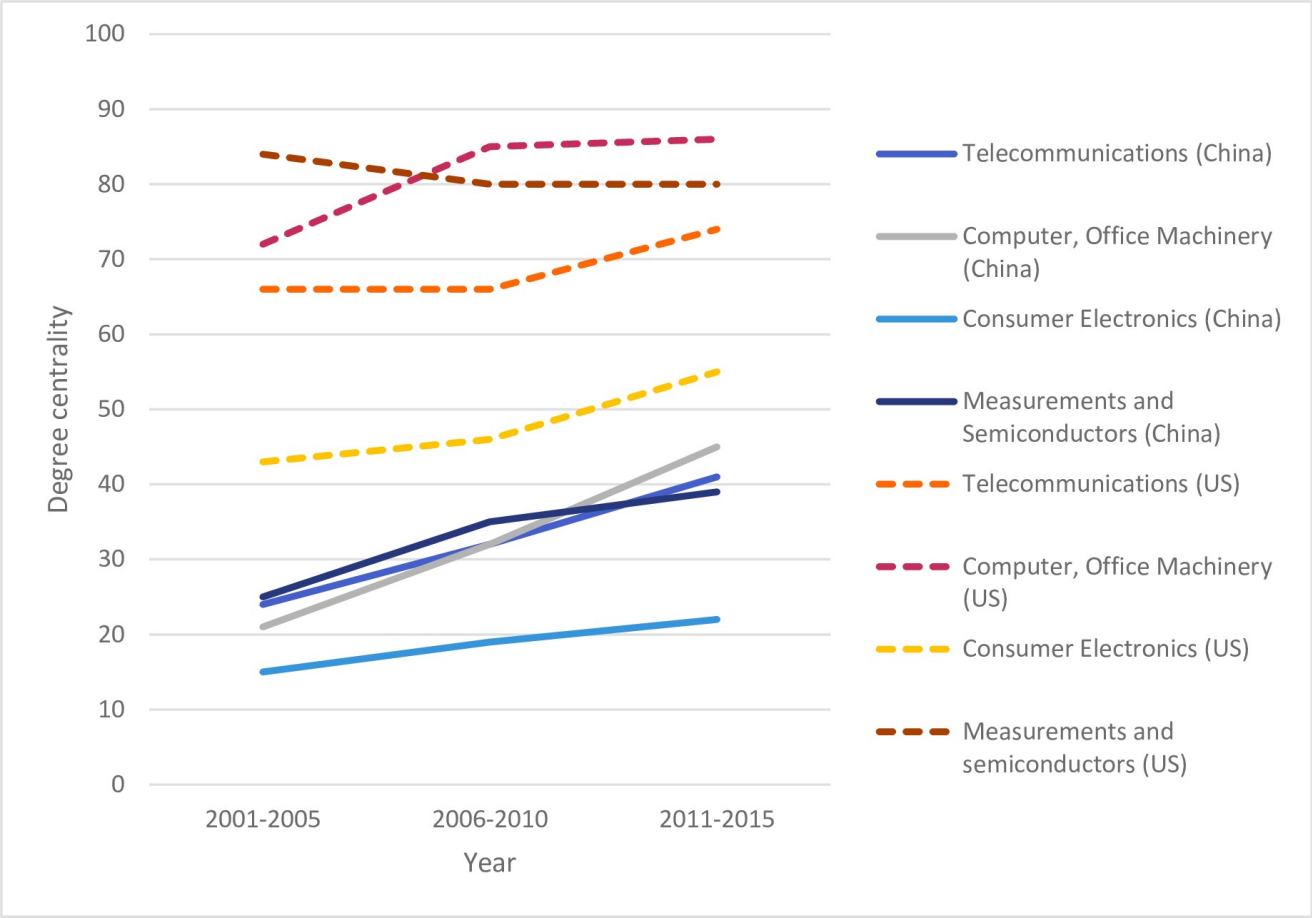
Degree centrality of US and China in the global ICT network (2001–2015).

Fig 3 compares the development in degree centralities of China over the observed time periods with the US. It shows that the US is still ahead of China in all sub-industries, but the catching-up process of China is well recognizable. China more than doubled its degree centrality for three sub-industries, while the US largely stagnated, though of course at a very high level (with the exception of *Consumer Electronics*). Note in this context, that China has overtook the US as the world’s largest patent producer in ICT in the year 2015 (in absolute numbers, not per capita [44]), with an enormous growth just very recently over the past decade. This explains, on the one hand, why China is still behind the US in terms of network centrality, but, on the other hand, suggests that this catching-up process is not yet finished.

It is worth noting in this context that the development of China in terms of patenting, and the influence of Chinese innovation and science policy on patenting has been discussed critically in the literature (see e.g. [57–59]). It is stressed that the growth in patenting has been strongly subsidized by Chinese government (directly and indirectly by different incentives), leading to this high patenting propensity of actors in the Chinese innovation system. It is also argued that the patent growth does not necessarily translate into innovation as the quality of the patents is assumed to be relatively low. However, the first rank in global ICT patenting [44] already refers to PCT patents only, i.e. those patents that have global significance by definition [50]. Moreover, in the ICT sector there are a number of Chinese firms that meanwhile take a strong global market position that cannot be reached without being highly innovative. In this sense, the Chinese government seems to be aware that the pure quantity of patents is not a policy goal ensuring the sustainable development of the Chinese economy and has put parallel emphasis on strengthening the Chinese research and innovation ecosystem as a whole, in particular in the education sector (see e.g., [60]).

## 4 Discussion and concluding remarks

Today it is widely recognized that R&D collaboration networks are a central element of modern innovation processes, in particular in times of increasing globalization and increased risk and uncertainty. Such networks nowadays increasingly spread within and across country borders, well documented in the literature of R&D internationalization (see, e.g., [22]), enabling researching and innovating actors to access relevant, globally dispersed knowledge sources, reflected by increased international scientific (e.g. co-publications), technological (e.g. co-patenting) or project-based joint R&D endeavors. While we can observe a growing body of empirical literature investigating structures and dynamics of international R&D collaboration networks at an aggregated level of countries or regions (see, e.g., [12, 17, 19]), there are only a few works exploring systematically such networks for specific scientific or technological fields, such as the ICT sector. This has been a significant research gap, given that ICT–considered as general-purpose technology influencing value added across almost all economic sectors (see [38])–is characterized by fast innovation cycles, and therefore specifically relevant in a network context, given the ability of such networks to reduce risks and uncertainties in the innovation process.

The purpose of this study was to address this research gap and to systematically characterize the structure and dynamics of global R&D collaboration networks in ICT by analyzing a large-scale dataset on cross-country co-patent activities in ICT, reflected in patents featuring at least two inventors from two different countries, accordingly signaling R&D collaboration crossing country borders. In its conceptual and theoretical approach, the study emphasizes the importance of networks for innovation as well as the significance of the ICT sector for economic growth. In our methodological approach, we employ a social network perspective for analyzing the structure and dynamics of the networks under consideration, using co-patents viewed as proxy for the cross-country network of R&D collaboration in ICT for the time periods 2001 to 2005, 2006 to 2010 and 2011 to 2015, and disaggregating the ICT sector in four relevant subsectors. We have visualized the networks for the observed time periods to explore its structural dynamics and have calculated different global and local indicators from Social Network Analysis (SNA) enabling us to shed original light on global network dynamics, on the one hand, and the role of different countries, in particular China and the US, on the other hand.

The empirical analysis has produced a number of highly interesting insights that can be well related to theoretical considerations ongoing in the literature on R&D collaboration networks (see [4]), but also to policy debates, e.g. about the catching-up process of China at a global scale. As we would expect from theoretical assumptions in innovation research, but also from network theory (see Section 1), we indeed can observe that international R&D collaboration networks in ICT have become much larger in magnitude (more countries but also more inter-linkages), less centralized and more densely connected. This transformation–also observed in the literature at an aggregated level of regions or countries, or for other fields (see e.g. [6, 17, 19])–is even more pronounced in the ICT sector. It seems that–on the one hand–the necessity to cope with the increasing complexity in R&D–leading to higher uncertainty and risks–‘forces’ a higher number of innovating actors to collaborate in general, and increasingly also at an international level (see e.g. [61]). On the other hand, network structural mechanisms are a stake, e.g. preferential attachment and triadic closure that are often observed for all kind of dynamic social systems, leading to a more diversified and less centralized network structure over time. In view of the four subsectors in ICT, the strongest development towards larger, less central and more connected networks can be observed in the *Computers*, *Office Machinery* and in the *Telecommunications* sub-sectors. Less changes are noticeable in *Measurements and Semiconductors* and *Consumer Electronics*.

Turning to the local, country-centric perspective, the results show that the US is continuously holding the most central network position, followed by larger European countries and new emerging ones like India, Israel, and in particular China coming closer to the center of the network. However, at the local level, there are more remarkable changes when considering the different sub-industries of the ICT-sector. Most importantly, we can observe an evolution from a bipolar to a tripolar world, as previously shown for scientific collaborations [17], where the powerful, well connected position of the US weakens relatively compared to other, increasingly connected countries, most importantly China that has tremendously increased its centrality in the subsectors *Computers and Office Machinery*, *Telecommunications* and, *Measurements and Semiconductors*. Being aware that China has already surpassed the US in total patenting in ICT in 2015, this may in principle not be that surprising. However, some scholars have raised doubts whether China is able and/or willing to collaborate internationally to the same extent as reflected by their patenting intensity, pointing to the potentially lower quality of Chinese patents and to the somewhat non-transparent effects of governmental supports for patenting (see e.g. [62]). In that sense, the catching-up of China from a network perspective as observed in this study is quite illuminating and partially challenges doubts about the quality of Chinese patents, at least for the ICT sector, since international collaborations usually tend to be subject to higher rather than lower quality of R&D activities, given that foreign partners would not engage in low quality R&D collaboration.

In light of these insights and in a policy context, it is worth reflecting on the determining factors for the rise of China’s ICT sector. *Firstly*, China has put immense emphasis on fostering R&D and strengthening the Chinese innovation system as a whole, and in stimulating national and international R&D collaboration in particular (see [16] for a review); these policies comprise a strong development of the higher education sector, with a strong focus on natural sciences and engineering [60]. This has not only improved the own innovation capability of China, but also increased its absorptive capacity, i.e. the ability to absorb technological knowledge from the many foreign firms investing in China which has played a major role in the past two decades [20]. *Secondly*, and as a major complementary effect to the first one, China follows a well-directed government-controlled investment plan of economic development with the ambition to build up the global leadership in ICT. China provides some of the leading digital marketplaces and is home to a third of all unicorn startups–which are startup companies with a current value of more than $1 billion before going public or the investors exit–worldwide [63]. In this context, China has also immensely advanced in e.g. artificial intelligence and related applications, blockchain technologies and quantum-computing. Furthermore, there are many successful, meanwhile large-scale Chinese companies in ICT like Huawei (the currently largest patent applicant in ICT worldwide), Alibaba or Tencent, acting at a global scale [64].

In a wider sense, some specific policy conclusions come to mind when recapitulating the results. *First*, acknowledging that strong ICT capabilities have positive effects on economic growth and productivity (see [38]), it is recommended to design policies–ranging from effective education systems, targeted R&D subsidies to specific incentive policies–that support and stimulate in a sustainable manner the ability to participate in these global ICT networks (see [37, 32]). Since the innovation capabilities in ICT are increasingly driven by taping international knowledge sources via international R&D collaborations, loosing connection and influence in such networks will have negative economic effects in the long-term. *Second*, in light of the economic productivity effects of ICT, policy authorities should set agendas fostering the translation of R&D activities into concrete businesses and products.

While the study produces interesting original results, we have to consider its limitations that at the same time pave the way for a future research agenda. *First*, using co-patents as indicators limits the comprehensiveness of the study to a specific type of R&D collaboration. It would be interesting whether we can find similar dynamics using indicators on scientific R&D collaborations as reflected in co-publications, or other forms of joint R&D. *Second*, monitoring the ongoing dynamics in this important, generic industry is crucial, in particular in light of the observed catching-up processes of Asian countries, mainly China. Moreover, it would be quite interesting to compare with other key technological fields, such as biotechnology, pharmaceutics, photonics or advanced manufacturing. For instance, for the Chinese case, it could be assumed that the catching-up is lower in other fields than in ICT, given the longer innovation cycles e.g. in pharmaceutics, and the strong focus on ICT in Chinese innovation policy. *Third*, the study takes a descriptive perspective and does not empirically explain emerging network structures, i.e. its determining factors and its relation to other economic variables of interest, such economic productivity. Accordingly, a more systematic and statistical investigation of the drivers and determinants for the observed network dynamics has a top priority for future research (see, e.g. [33]), but also the relation of observed R&D collaboration network structures and economic productivity growth patterns at country level. This needs a move from descriptive to explanatory network analytic approaches, for instance by using exponential random graph or spatial interaction models to estimate country-specific relational factors influencing the dynamics of the observed ICT R&D collaboration networks.

## Supporting information

S1 Appendix (DOCX)
