# Supplementary material for: The dynamics of global R&D collaboration networks in ICT: Does China catch up with the US?
Source: PLoS One. 2020 Sep 1;15(9):e0237864. doi: 10.1371/journal.pone.0237864 (PMC7462303; doi:10.1371/journal.pone.0237864)
Supplement: S1 Appendix — (DOCX) [file pone.0237864.s001.docx]

**Appendix A: Data extraction and preparation**

This appendix provides a detailed description of the processes of data extraction and preparation, specifically the assignment of patents to ICT and respective technological subfields.

1. *Data extraction*

Our sample comprises 77 thousand co-patents applied for in the years 2001-2015; specifically, cross-country co-patents that have at least two inventors located in different countries. Patents are extracted from the RISIS-Patent database (https://rcf.risis2.eu/dataset/5/metadata) based on PATSTAT by the European Patent Office (EPO). Specifically, we rely on patents classified according the international Patent Cooperation Treaty (PCT). With this, we restrict the sample to globally relevant patents, on the one hand, and avoid bias due to differences related to country-specific patent offices’ behaviour, on the other hand (see [45]).

1. *Delimitation of ICT patents*

In the delimitation of ICT patents, we follow the standard OECD classification that assigns patents to ICT technological fields based on the International Patent Classification (IPC) system [34]. The OECD [34] classification distinguishes the ICT sector in four subclasses also investigated in this study; these are *Computers and Office Machinery*, *Consumer Electronics*, *Telecommunications* and *Other ICT*. We have chosen to re-label the class *Other ICT* to *Measurements and Semiconductors* to make it more tangible as these are the most prevalent technologies in this residual group. Note that recent revisions of this classification (see [34]) introduced a finer grained sub-structure that can again be aggregated to these four fields, however, did only change marginally as what concerns ICT as a whole. Hence, in order not to increase the manifoldness in terms different sectors and analytical possibilities too much, we stay at this level of ICT as a whole and these four technological subfields.

These four ICT sectors are identified based on a comprehensive allocation of 4-digit IPC codes developed by the OECD that relies on a content examination of IPC classes and subclasses, as well as keyword-based searches performed on the full text of the patent [34]. Hence, patents in these four fields are split based on selected IPC patent classes, as presented in Table A1.

1. *Data preparation*

The study uses cross-country co-patents, i.e. patents that have at least two inventors located in different countries. Extracted ICT patents are assigned to countries based on the inventors’ places of residence. If one patent has more than one inventor in different countries, the patent is counted for each of those countries. Hence, when constructing the cross-country co-patent networks, we count each country combination as one link (intra-country co-patents are not accounted for). This results in a *n*-by-*n* adjacency matrix, where *n* denotes the number countries. The elements of this matrix correspond to the number of co-patents between countries.

**Table A 1. IPC patent classes of ICT patents**

| **Telecommunications** | |
| --- | --- |
| G01S | Radio direction-finding; Radio navigation; etc. |
| G08C | Transmission systems for measured values, control or similar signals |
| G09C | Ciphering or deciphering apparatus for cryptographic or other purposes involving the need for secrecy |
| H01P | Waveguides; resonators, lines or other devices of the waveguide type |
| H01Q | Aerials |
| H01S3 | Lasers |
| H01S5 | Semiconductor lasers |
| H03B | Generation of oscillations |
| H03C | Modulation |
| H03D | Demodulation or transference of modulation from one carrier to another |
| H03H | Impedance networks, e.g. resonant circuits; resonators |
| H03M | Coding, decoding or code conversion, in general |
| H04B | Transmission |
| H04J | Multiplex communication |
| H04K | Secret communication; jamming of communication |
| H04L | Transmission of digital information |
| H04M | Telephonic communication |
| H04Q | Selecting |
| **Consumer electronics** | |
| G11B | Information storage based on relative movement between record carrier and transducer |
| H03F | Amplifiers |
| H03G | Control of amplification |
| H03J | Tuning resonant circuits; selecting resonant circuits |
| H04H | Broadcast communication |
| H04N | Pictorial communication |
| H04R | Loudspeakers, microphones, gramophone pick-ups or like acoustic electromechanical transducers, etc. |
| H04S | Stereophonic systems |
| **Computers and Office Machinery** | |
| B07C | Postal sorting |
| B41J | Typewriters; selective printing mechanisms |
| B41K | Stamps; stamping or numbering apparatus or devices |
| G02F | Devices or arrangements, the optical operation of which is modified by changing the optical properties |
| G03G | Electrography; electrophotography; magnetography |
| G05F | Systems for regulating electric or magnetic variables |
| G06 | Computing; calculating; counting |
| G07 | Checking-devices |
| G09G | Arrangements or circuits for control of indicating devices using static means to present variable information |
| G10L | Speech analysis or synthesis; speech recognition; speech or voice processing; etc. |
| G11C | Static stores |
| H03K | Pulse technique |
| H03L | Automatic control, starting, synchronisation, or stabilisation of generators of electronic oscillations |
| **Measurements and Semiconductors (Other ICT)** | |
| G01B | Measuring length, thickness; measuring angles; measuring areas; etc. |
| G01C | Measuring distances, levels or bearings; surveying; navigation; gyroscopic instruments; etc. |
| G01D | Measuring not specially adapted for a specific variable; etc. |
| G01F | Measuring volume, volume flow, mass flow, or liquid level; metering by volume |
| G01G | Weighing |
| G01H | Measurement of mechanical vibrations or ultrasonic, sonic or infrasonic waves |
| G01J | Measurement of intensity, velocity, spectral content, etc. of infra-red, visible or ultra-violet light etc. |
| G01K | Measuring temperature; measuring quantity of heat; thermally-sensitive elements |
| G01L | Measuring force, stress, torque, work, mechanical power, mechanical efficiency, or fluid pressure |
| G01M | Testing static or dynamic balance of machines or structures |
| G01N | Investigating or analysing materials by determining their chemical or physical properties |
| G01P | Measuring linear or angular speed, acceleration, deceleration or shock; etc. |
| G01R | Measuring electric variables; measuring magnetic variables |
| G01V | Geophysics; gravitational measurements; detecting masses or objects; tags |
| G01W | Meteorology |
| G02B6 | Light guides; structural details of arrangements comprising light guides and other optical elements |
| G05B | Control or regulating systems in general; functional elements of such systems |
| G08G | Traffic control systems |
| G09B | Educational or demonstration appliances; models; planetaria; globes; maps; diagrams |
| H01B11 | Communication cables or conductors |
| H01J | Electric discharge tubes or discharge lamps |
| H01L | Semiconductor devices; electric solid state devices |

**Appendix B: Global R&D collaboration networks in four ICT subsectors (2001-2005, left; 2011-2015, right)**


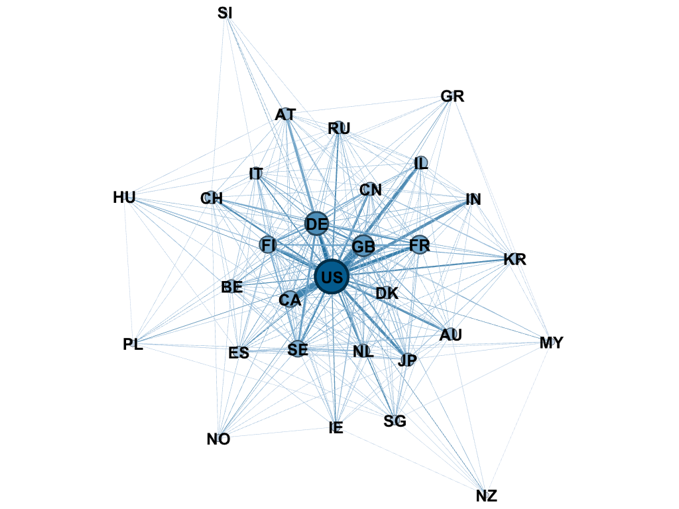

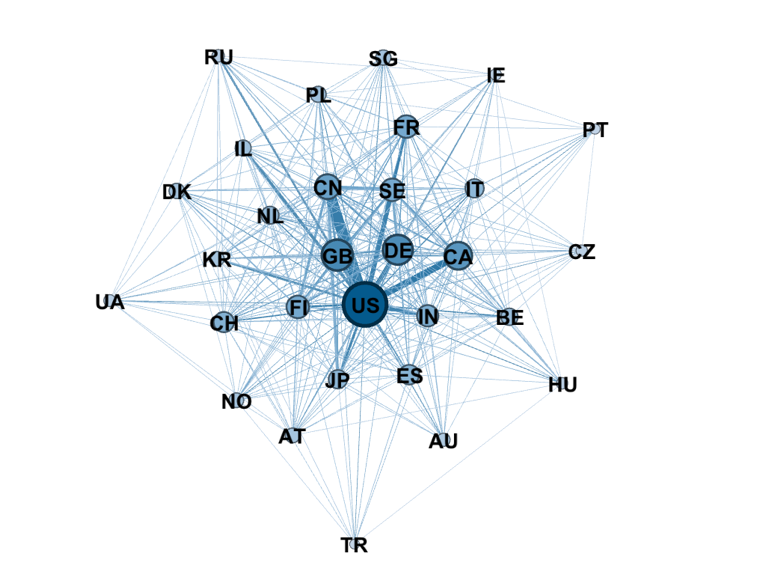
A: Telecommunications


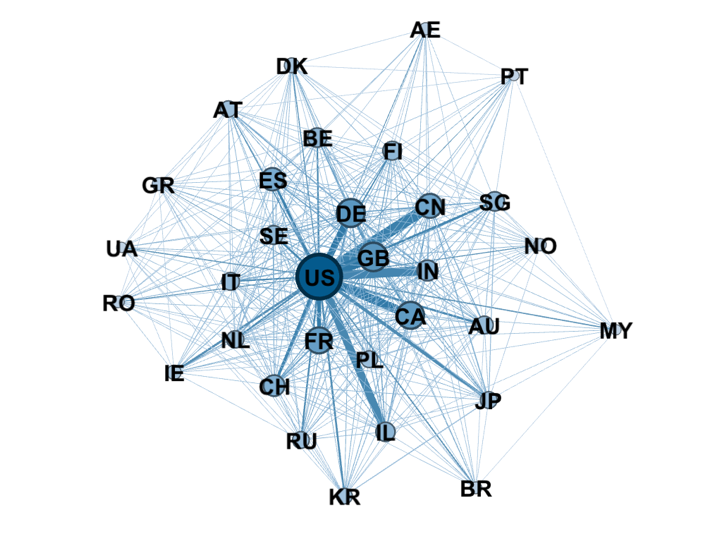

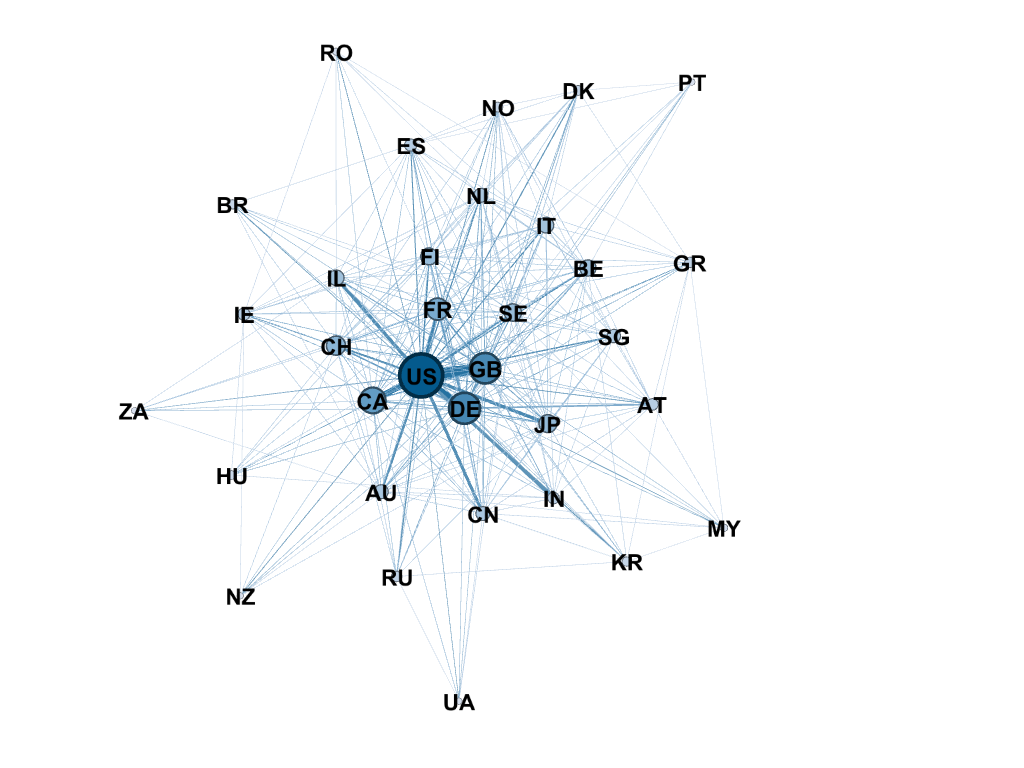
B: Office, Computer Machinery


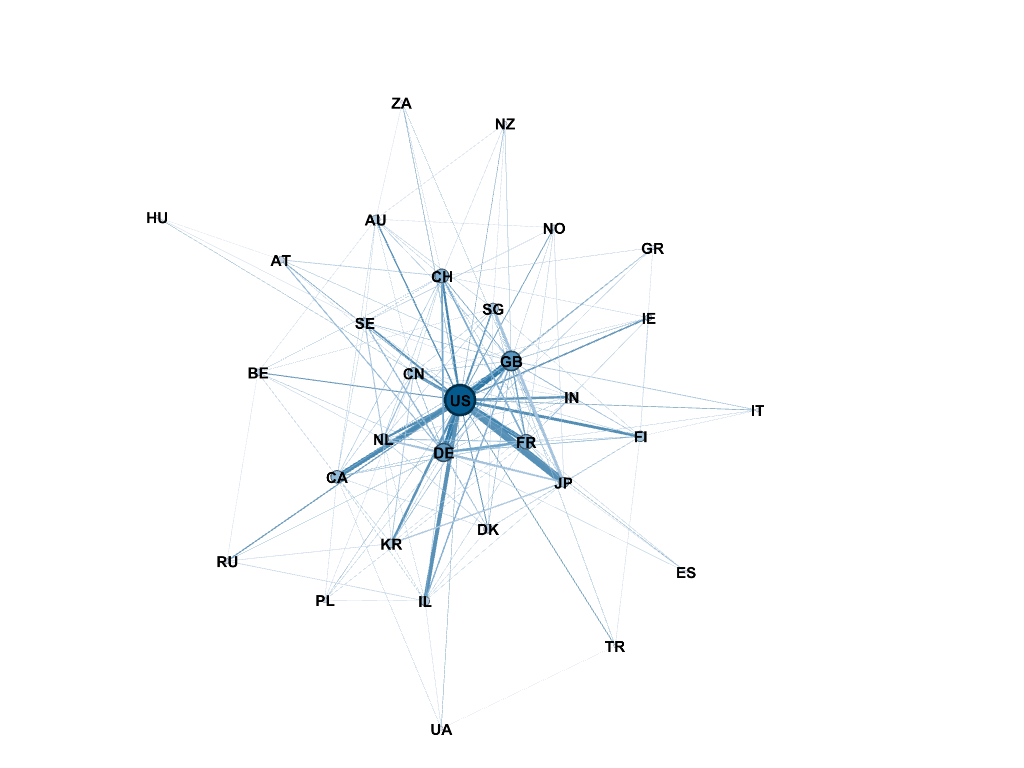
C: Consumer Electronics (2001-2005, left; 2011-2015, right)


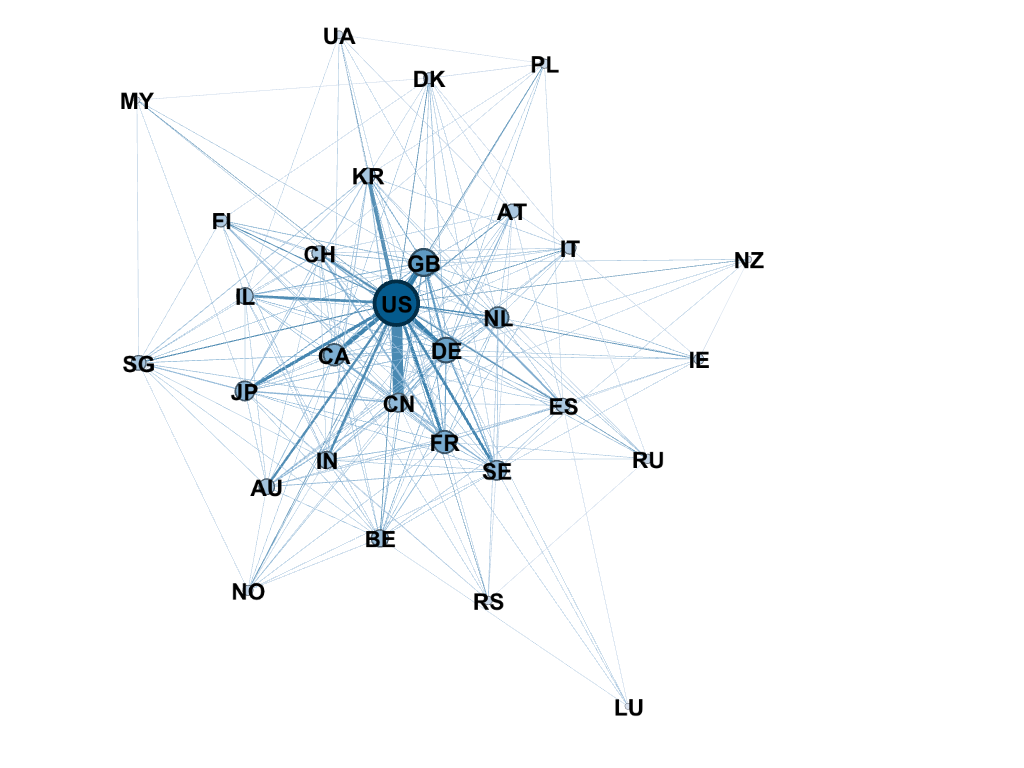


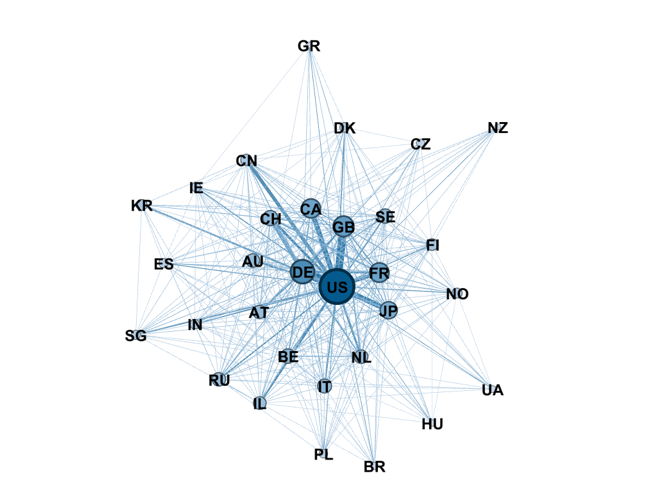

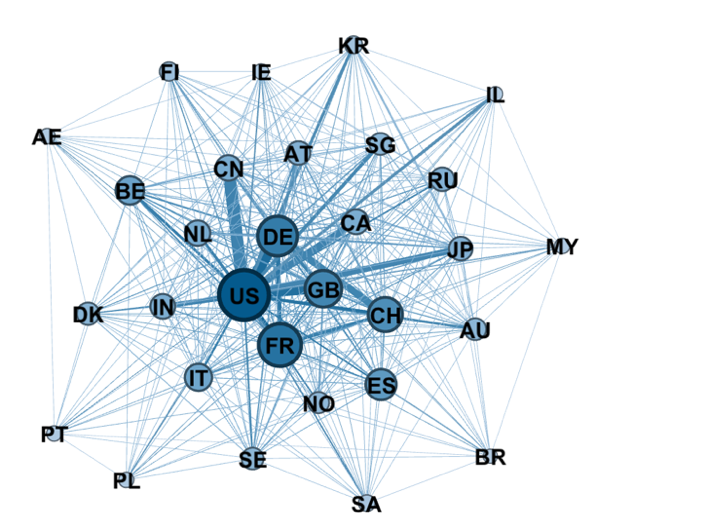
D: Measurements and semiconductors

*Notes: Country codes given in Appendix C*

**Appendix C: Country names and Alpha-2 code elements (ISO, 2020)**

| **Country** | **Alpha-2 code** |
| --- | --- |
| Algeria | DZ |
| Australia | AU |
| Austria | AT |
| Belgium | BE |
| Brazil | BR |
| Canada | CA |
| China | CN |
| Czechia | CZ |
| Denmark | DK |
| Finland | FI |
| France | FR |
| Germany | DE |
| Greece | GR |
| Hungary | HU |
| India | IN |
| Ireland | IE |
| Israel | IL |
| Italy | IT |
| Japan | JP |
| Korea, Republic of | KR |
| Luxembourg | LU |
| Malaysia | MY |
| Netherlands | NL |
| New Zealand | NZ |
| Norway | NO |
| Poland | PL |
| Portugal | PT |
| Romania | RO |
| Russian Federation | RU |
| Singapore | SG |
| Slovenia | SI |
| South Africa | ZA |
| Spain | ES |
| Sweden | SE |
| Switzerland | CH |
| Thailand | TH |
| Trinidad and Tobago | TT |
| Turkey | TR |
| Ukraine | UA |
| United Arab Emirates | AE |
| United Kingdom of Great Britain and Northern Ireland | GB |
| United States of America | US |
